# Impact of land use and land cover change on land degradation in rural semi-arid South Africa: case of the Greater Sekhukhune District Municipality

**DOI:** 10.1007/s10661-023-11104-0

**Published:** 2023-05-23

**Authors:** M. J. Kgaphola, A. Ramoelo, J. Odindi, J-M. Mwenge Kahinda, A. R. Seetal, C. Musvoto

**Affiliations:** 1 CSIR, P.O. Box 395, Pretoria, 0001 South Africa; 2 School of Agricultural, Earth and Environmental Sciences, University of KwaZulu-Natal, Scottsville, Pietermaritzburg, 3209 South Africa; 3 Centre for Environmental Studies, Department of Geography, Geoinformatics and Meteorology, University of Pretoria, Pretoria, South Africa

**Keywords:** Land use land cover changes, Land degradation, NDVI, Climate variability, Land management

## Abstract

In semi-arid regions, interactions between biophysical and socio-economic variables are complex. Such interactions and their respective variables significantly alter land use and land cover, degrade landscape’s structure, and impede the efficacy of the adopted land management interventions. This scenario is particularly prevalent in communal land tenure system or areas managed by a hybrid of traditional and state led institutions. Hence, this study sought to investigate the impacts of land use and land cover changes (LULCCs) on land degradation (LD) under communal rural districts, and the key drivers of habitat fragmentation in the Greater Sekhukhune District Municipality (GSDM), South Africa. The study used the wet and dry season multi-temporal remotely sensed image data, key-informant interviews, and workshop with tribal council to determine the major drivers of LULCC and LD. Results revealed that mines and quarries, subsistence and commercial cultivation, and thicket/dense bush LULCs declined significantly during the study period. These LULCs mostly declined in wet season, with loss in vegetation cover highly prevalent. Specifically, the highest conversions were from shrub/grassland to bare soil, thicket/dense bush to shrub/grassland, and shrub/grassland to residential, respectively. Generally, LULCC affected vegetation productivity within the study area, with increased negative NDVI values observed during the dry season. The findings from key informants and the tribal council workshop emphasized that soil erosion, abandonment of cropland, and injudicious land use (i.e. overgrazing and consequent bush encroachment) have severely degraded the land. The study also established that the degrading land can be attributed to the weakening local communal land management system, particularly the weakening tribal councils. The study recommends an urgent need for collaborative (i.e. government, tribal authorities, and land users) land management through designing relevant multi-stakeholder LD mitigation measures.

**Supplementary Information:**

The online version contains supplementary material available at 10.1007/s10661-023-11104-0.

## Introduction

Monitoring anthropogenic land use and land cover changes (LULCC) is critical for understanding the interactions of human activities with the environment at the local, regional, and global scales (Kuldeep & Kamlesh, 2011). Whereas humans have been deriving livelihoods from the natural environment for centuries, recently, the extent and intensity of uses have increased significantly. The expansion of infrastructure and agriculture necessitated by the ever-increasing population growth has quickened the pace of landscape transformation and degradation (Hooke et al., 2012). Land degradation affects 70% of drylands in South America, Asia, and Africa (Barbier & Hochard, 2016). In rural areas of the developing world, land degradation has become increasingly complex and severely affects rural livelihoods (Shafri et al., 2007; Bai et al., 2008). Hence, it is crucial to assess and monitor the impacts of LULCC to understand how they affect landscape productivity and sustainability (Gonzales Inca, 2009; Ibrahim et al., 2015).

Land is a crucial natural resource made of soil, water, and the associated flora and fauna (land cover). Anthropogenic land uses have changed land cover and rapidly and extensively disrupted ecosystems (Watson et al., 2014) and the services they provide. The demand for and unsustainable use of natural resources has intensified and changed land covers, severely degrading the structure, and functioning of ecosystems. Land conversion through injudicious land use practices such as unsustainable wood harvesting, overstocking, overgrazing, and agricultural intensification on arable lands and steep slopes accelerates the loss of ecosystem services and the land degradation process in arid and semi-arid regions (Mirzabaev et al., 2015).

Land degradation has been defined as the long-term decline of the ecosystem function and productivity arising from disturbances from which the landscape cannot recover unaided (Bai et al., 2008). The United Nations Convention to Combat Desertification (UNCCD) was established to halt land degradation in 1994 (UNCCD, 1994). However, despite the almost 30-year long endeavours throughout the globe, the situation has worsened. In 2012, the Land Degradation Neutrality (LDN) concept emerged from the UN Conference on Sustainable Development (Rio + 20). The aim of LDN is to meet future food and fuel demand without further degrading the finite land resource base (UNCCD, 2014). In this regard, three land degradation indicators (land use and land cover change, land productivity, and carbon stocks) are recommended to track progress towards LDN (UNCCD, 2016a). These indicators address ecosystem changes, ecosystem health. and habitat fragmentation arising from land use and other factors (UNCCD, 2016b).

In South Africa, land use is a particularly complex issue, partly due to the physical planning policies of the previous political dispensation. Under the 1913 Land Act, 13% of the country’s land was held in trust as homelands (currently communal areas) in which 50% of the black population (nearly 3.5 million people) were resettled (Fox & Rowntree, 2001). Communal areas have a long history of environmental and state governance neglect; hence, the constrained high densities of people and livestock resulted in rampant land degradation (Hoffman et al., 1999; Meadows et al., 2002; Ross, 1999). Currently, land conflicts arising from ownership, access, and rights (i.e. land tenure) have contributed to unsustainable land use practices that include overgrazing and excessive wood harvesting, which have in turn led to soil erosion and the invasion of unpalatable plants (Duraiappah et al., 2000). Hence, many communal areas of the North-West, Northern Cape, Eastern Cape, Mpumalanga, and the Limpopo provinces are severely degraded (Department of Environmental Affairs and Tourism (DEAT), 2005; Dubovyk et al., 2015; Graw et al., 2017).

Several studies have adopted remotely sensed data to assess and monitor the spatial and temporal variability of landscape transformation and LULCC (Karnieli et al., 2008; Mashame & Akinyemia, 2016; Match et al., 2012; Ganasri & Dwarakish, 2015). Conventional LULC monitoring techniques such as field surveys, review of existing literature, map interpretation, and ancillary data analysis are often tedious, time-consuming, and costly (Xie et al., 2008). On the other hand, satellite-based remotely sensed data can be used to quantify, map, and detect patterns of LULCC due to their reliable accuracy, a digital format suitable for computer processing, repetitive data acquisition, and the possibility to access remote areas at different seasons (Lu & Weng, 2007; Chen et al., 2005; Rahman et al., 2011). The Landsat programme, for instance, provides the longest medium spatial resolution satellite data since its launch in 1972 and has been widely used to compute LULC (Pandey et al., 2021; Rocchio et al., 2005). Since detecting LULCs requires accurate and up-to-date information regarding initial and final land LULCs (i.e. from-to), sensors like the Landsat series, with a rich archival data and relevant image analysis algorithms, are invaluable (Giri et al., 2005).

Several studies have explored the influence of biotic, abiotic, and environmental impacts on LULCC at regional scales (James et al., 1999; Leidinger et al., 2017; Ludwig et al., 2001). At local scales (i.e. district level or lower), most studies have considered specific LD degradation variables like deforestation, soil erosion, bush encroachment, and plant species invasion (Match et al., 2012). This study adopts a holistic view of LD on all LULC units under dual management system (i.e. traditional and state led local governance) within the study area. Specifically, this study seeks to provide detailed information on LD in communal areas and its implication on grazing and crop farming systems. Whereas previous studies have linked LULC to LD (e.g. Hoffman et al., 1999 and Meadows et al., 2002), there is a dearth of literature on such linkages in communal rural districts within semi-arid landscapes. Specifically, land degradation under various management and land tenure regimes as well as key drivers remains largely unexplored (Rowntree et al., 2004).

Understanding LULC dynamics and LD is critical for promoting sustainable natural resources use and rural livelihoods under a dual land management system (Meshesha et al., 2016). In this regard, rates and predictors of habitat conversion, particularly in a fragile communal semi-arid landscape, are fundamental for designing policies and developing effective strategies for sustainable natural resource use and management. Hence, the objectives of this study were to, firstly, assess the evolution of LULCC from 1990 to 2019, secondly, quantify and analyze land use and land cover changes, and, thirdly, identify driving factors and impacts of LULCC on land degradation within the Greater Sekhukhune District Municipality, South Africa.

## Materials and methods

### The study area

**Fig. 1:**
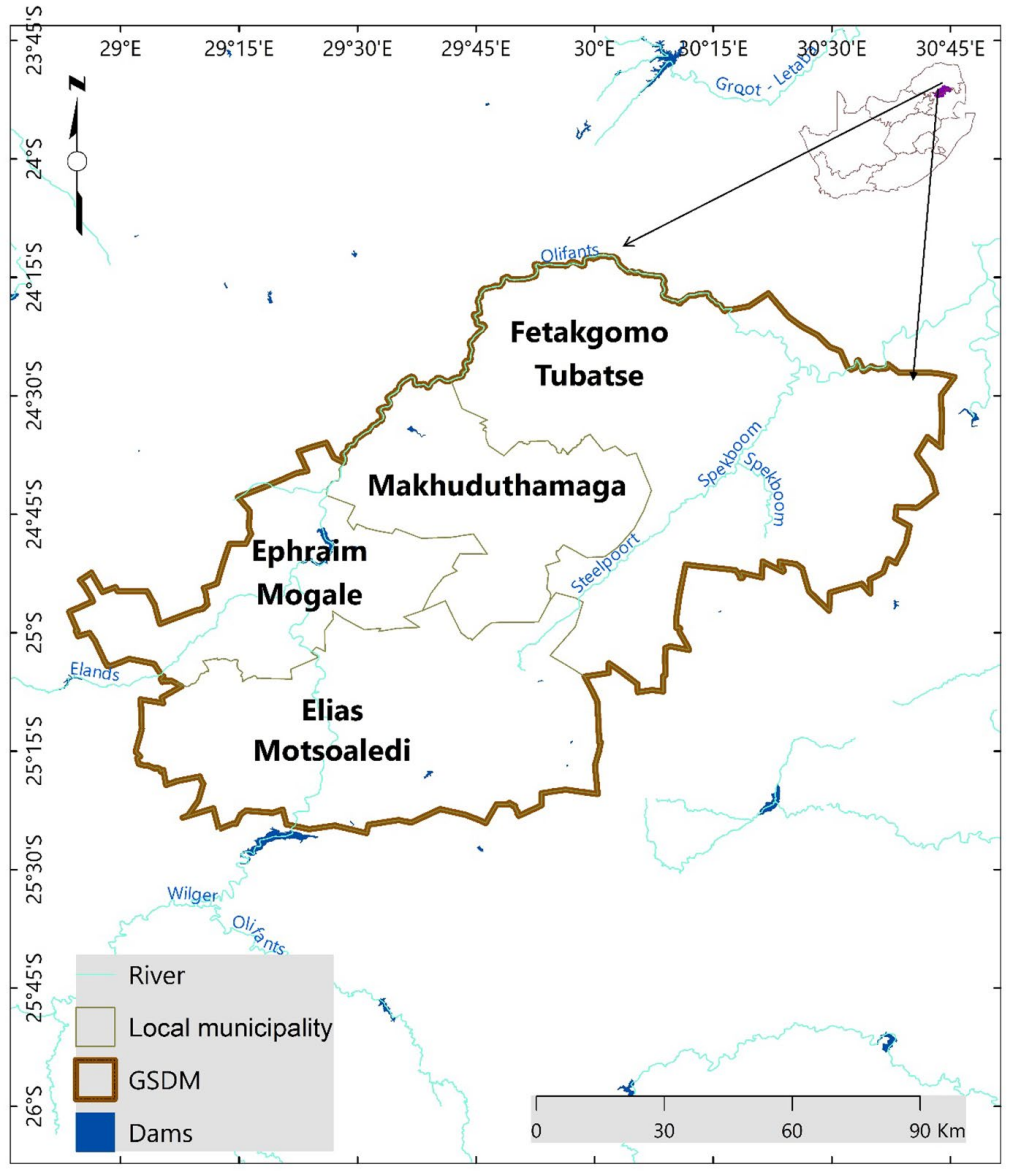
The Greater Sekhukhune District Municipality (GSDM)

The Greater Sekhukhune District Municipality (Fig. 1) is in the Limpopo province, the north most part of South Africa (24° 5′ 10″ S, 25° 21′ 27″ S and 29° 3′ 40″ E, 30° 44′0.30″ E). The district has four local municipalities (Elias Motsoaledi, Ephraim Mogale, Makhuduthamaga, and Fetakgomo Tubatse) covering approximately 1,352,800 Ha. The total population is approximately 1,090,424, mainly living in rural communal areas (Statistics South Africa, 2016).

The district is in a semi-arid environment, with an average annual rainfall of ± 560 mm and average summer temperatures of approximately 23 °C (Stronkhorst et al., 2009). The geology dominating the area is ultramafic substrates, known as serpentine soils of the Rustenburg-layered suite bushveld complex (Gourmelon et al., 2016). These soils are mainly characterized by low nutrient availability (e.g. nitrogen, potassium, and phosphorus) and high concentrations of heavy metals (e.g. cadmium, zinc, and nickel) (Gourmelon et al., 2016). Topography is characterized by undulating hills ranging from hilly to mountainous, with an average altitude of 494 m above sea level (GSDM IDP, 2019). The biome of the district is savannah dominated by natural grassland thicket, bushveld, bush clumps, and high fynbos covers (Acocks, 1988).

Agriculture is important in the district, with commercial farming accounting for 7.7% and subsistence farming 18.1% of the land use in the district (GSDM IDP, 2020). However, most of the croplands have been abandoned and water scarcity, land conflicts, a high number of land claims, and inappropriate infrastructure and services threaten future viability of agriculture in the area (Mpandeli et al., 2015; GSDM IDP, 2019). Unlimited access to communal grazing and lack of fencing in fields have intensified land degradation due to low herbaceous basal cover (Shackleton et al., 2013; Hoffman & Ashwell, 2001).

### Methods

This study used a mixed-methods approach that integrates quantitative and qualitative techniques to better understand LULCC, LD, their drivers, and impacts. Using remote sensing (RS) techniques and Geographical Information Systems (GIS), seasonal LULC images were classified from Landsat Thematic Mapper (TM) and Operational Land Imager sensor (OLI) scenes. The impacts and drivers of LULCC on LD were analyzed using change detection of LULC and key-informant interviews.

### Data collection

#### Remotely sensed data

Given that long-term monitoring is necessary to determine LD, Landsat satellite images with 30 m spatial resolution were obtained from the United States Geological Survey (USGS) Global Visualization Viewer (http:// glovis.usgs.gov/) dataset for the years 1990, 1995, 1999, 2005 and 2010, 2015, 2019 (30 years at 5-year intervals). To capture and assess seasonal climatic variability and change over the Limpopo province, seasonal images were selected from wet and dry seasons. The rain peaks in January and February; hence, these months were used for wet season assessments as there is ample agricultural and vegetation growth and filled water bodies, while May to August months were used to represent the driest months (Mpandeli et al., 2015). Good-quality images with less than 10% cloud cover were collected. Only one image had cloud cover over 10% (cloud cover of 14%), which did not compromise the results. Due to data availability in some years, alternative closest possible images were used.

#### Key-informant interviews and workshops with tribal councils

Snowball sampling was used to select key informants based on their skills and experience in using and managing grazing land, cropping, fuelwood and rangeland, water, and soil resources in the GSDM (Payne & Payne, 2004). A semi-structured questionnaire was used to interview 11 key informants from the Limpopo Department of Agriculture and Rural Development (LDARD) based in the study area (Table 1). The key informants, interviewed individually, included natural resource managers, crop and animal production, and extension services personnel within each local municipality.

**Table 1:** Key informants interviewed in GSDM per local municipality and years of experience working in the municipality and field

Local municipality	Key informant	Field of expertise	Years of experience
Fetakgomo Tubatse	1	Extension services	40
	2	Natural resource management	13
	3	Natural resource management	14
	4	Natural resource management	12
	5	Crop production	14
Makhuduthamaga	6	Natural resource management	12
	7	Crop production	24
Elias Motsoaledi	8	Animal production	10
Ephraim Mogale	9	Extension services	7
	10	Extension services	15
	11	Animal production	12

The semi-structured questionnaire was designed to acquire information on historical LULCC, physical factors, socio-economic profiles, and cultural data to determine LD driving factors and their impacts. Inputs were provided on the drivers of LULCC, grazing and rangeland management, and impact of LULC changes on land degradation experienced in the district over the past 30 years. The interviews also included information on laws and regulations that affect access to land, use, and impacts observed over the years.

Land in many parts of rural South Africa is controlled by traditional structures, with Traditional Authorities (TAs) (comprising a chief and their council) playing a key role in allocating land and decisions on land use (Musvoto et al., 2022). As part of understanding the drivers of LULCC and land degradation in GSDM, open informal discussion sessions were held with 17 Traditional Authorities, with questions focussing on their perceptions and experiences on land and natural resource-based activities, state of land and natural resources, land degradation (its causes, and impacts on land-based activities), current agricultural activities, and role of TAs in land management and mitigating land degradation.

### Data analysis

The following RS classification/mapping procedures were used: classification scheme development, image pre-processing, classification, validation, change analysis, and post-classification LULCC.

#### Classification scheme

Due to the diverse land cover types and land uses, it was necessary to identify and classify land according to its characteristics and use potential (Rhind, 1993). The South African national standard for Land Cover Classification System (Department of Rural development and Land Reform, 2019) was applied to map the existing LULC in the study area (Table 2). The broad hierarchical level 1 was applied on Landsat images to identify existing LULC. Furthermore, since the study investigated the impacts of LULCC on land degradation, levels 2 and 3 were applied to identify barren, cultivated, and residential land for a detailed mapping of these classes.

**Table 2:** LULC classes and their descriptions (Department of Rural development and Land Reform, 2019)

LCC level	Class name	Description
1	Shrub and grassland	Perennial grass, sparse trees, impoverished woodlands, very sparsely distributed, low-lying shrub species
1	Thicket/dense bush	Bush land, dense shrubs
1	Bare/exposed rock	Bare, exposed areas and transitional areas
1	Mines and quarries	Areas in which mining activities has been conducted. This includes both opencast mines and queries, surface infrastructure, mine dumps
1	Residential	Built-up areas used for residential (town or villages), commercial and services, and transportation
1	Water bodies	Water reservoirs and water channels. Includes all natural and artificial surface water
2	Commercial cultivation	Cultivated lands used primarily to produce rain-fed, annual crops or primarily to produce centre pivot/non pivot irrigated for commercial markets. Typically represented by large field units, often in dense local or regional clusters
2	Subsistence cultivation	Rain-fed, annual crops for local markets and/or home use. Small field units, often in dense local or regional clusters
3	Eroded land	Non-vegetated donga and gully features, typically associated with significant natural or man-induced erosion activities along or in association with stream and flow lines. The mapped extent of the dongas and gullies is represented by bare ground conditions in all, or the majority of the multi-date Landsat images used in the land cover modelling

#### Satellite image pre-processing

Satellite images were radiometrically and geometrically corrected and re-projected (Ganasri & Dwarakish, 2015). The Landsat images were geo-referenced to correct false changes in seasonal LULCC using the Georeferencing tool in ArcGIS 10.3. The images were also radiometrically corrected to improve their quality using the histogram equalization approach. Image colour was balanced using the colour corrector tool and first order dodging methods.

#### Image classification

The images were classified using the supervised classification scheme and the maximum likelihood classifier. The maximum likelihood algorithm was applied because it utilizes the mean, variance, and covariance of training site’s digital numbers (Sisodia et al., 2014). The classifier takes advantage of the probability of a pixel being a member of an information class in its decision making (see Eq. 1). This algorithm relies on the second-order statistics of the Gaussian probability density function model for each class (Ganasri & Dwarakish, 2015).

**Fig. 2:**
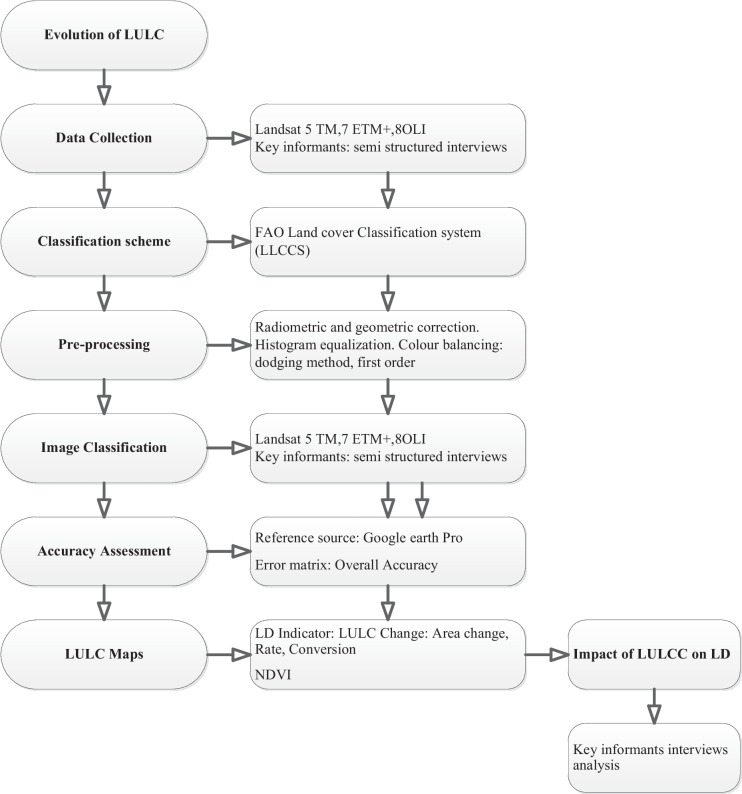
Flow chart of the methodology adopted the study

Supervised classification is an iterative process where collected training samples must be evaluated and edited as images are classified to increase accuracy (see Fig. 2). A minimum of 10 training areas were collected for each land class as recommended in the literature to adequately create signature files and classify images using the classifier (Meshesha et al., 2016). Then, training samples are re-evaluated, re-edited, and re-collected if training samples are inaccurate (Meshesha et al., 2016). High-resolution Google satellite images were used as secondary sources to improve classification accuracy (Cao et al., 2016; Kobayashi et al., 2014).

#### Accuracy assessment

One of the most popular accuracy assessment approaches is the error matrix (Foody, 2010). An error matrix is an effective way to represent accuracy in that the accuracies of each category are plainly described along with both the errors of inclusion (commission errors) and errors of exclusion (omission errors) present in the classification (Congalton, 1991). The overall accuracy (OA) indicates the total number of successfully identified samples in the classification relative to the total number of samples that occur in the classified image. Accuracy assessment was conducted using the ArcGIS software to produce an error matrix report with the OA and Kappa coefficient. The sampling strategy used was the equalized stratified random method selector that creates points that are randomly distributed within each class, where each class has the same number of points (Congalton, 1991). The number of random points was 10 times the number of test pixels for each class. Since there were nine land cover classes in this study, 30 test pixels for each land cover were randomly created resulting in a total of 270 test pixels to assess classification accuracy. High-resolution Google satellite images were used to collect reference points.

#### Change detection

As set out by the United Nations Convention to Combat Desertification (UNCCD) recommendation for tracking progress towards LDN (UNCCD, 1994; Orr et al., 2017), LULC change and NDVI (land productivity) were used as indicators for LD in this study. Landscape transformation provides a first indication of changing vegetation cover and habitat fragmentation (Cha et al., 2020; Orr et al., 2017). Land productivity captures changes in ecosystem functions and health (UNCCD, 1994). In this study, habitat fragmentation was monitored using the Temporal Image Differencing (TID) method to quantify changes on LULC classes. Temporal Image Differencing is a process whereby pixels from the first-date image are subtracted from those of the second-date image, thus generating a third image (Lillisand et al., 2014). Area change, land conversions, and rate of change (Eq. 1&2) are one of the many ways to study land cover change (Meshesha et al., 2016). Area change refers to the change in the extent of a certain type of land cover from the beginning to the end of the study period (Eq. 1) (Meshesha et al., 2016). Land conversion refers to the conversion of a type of land into other types at the beginning and end of the study period with a LULC transition matrix applied to determine and quantify the changes. Rate of change is the spatial transformation in relation to time, i.e. hectare per year per land class (Eq. 2). The following formulae were applied to determine LULC change:

1 $${C}_{e}= \frac{{T}_{a}\left({t}_{2}\right)-{T}_{a}\left({t}_{1}\right)}{{T}_{a}({t}_{1})}\times 100$$

where *C*_*e*_ is the percentage change in area extent; *T*_*a*_ is the total area; *t*_1_ is the initial time; *t*_2_ is the ending time.

2 $${C}_{r}=\frac{\left(\frac{ {T}_{a}\left({t}_{2}\right)-{T}_{a}\left({t}_{1}\right)}{{T}_{a}\left({t}_{1}\right)}\right)}{{t}_{2}-{t}_{1}}\times 100$$

where *C*_*r*_ represents the annual rate of change; *T*_*a*_ is the total area; *t*_1_ is the initial time; *t*_2_ is the ending time.

 199Vegetation change reflects effects of various factors including climate, abiotic environment, biotic interactions, and disturbance history. Vegetation production from time-series satellite images is one of the most useful indicators of LD at regional or global scales (Fensholt et al., 2013; Holm et al., 2003; Veron et al., 2006). In arid or semi-arid areas, NDVI is strongly correlated with above-ground net primary productivity (ANPP) (Huang & Kong, 2016); hence, it was used as a proxy for LD in this study. NDVI measures vegetation condition and its health and calculates the difference derived from visible and near-infrared portions of the electromagnetic spectrum (Wessels et al., 2004). NDVI is calculated using the formula below:

3 $$NDVI=\frac{NIR-RED}{NIR+RED}$$

where *NIR* is reflection in the near-infrared spectrum (nm); *RED* is the reflection in the red range of the spectrum by vegetation cover (nm).

The values range from − 1 to + 1, with high values representing healthy/active vegetation while non-vegetated surfaces such as water bodies and bare land/ rocks are represented by negative NDVI values (Wessels et al., 2004). NDVI was extracted on the wet and dry season Landsat images then Image Differencing was applied for every 5-year period using ERDAS Imagine 2018 software. NDVI change detection images and statistics were acquired using Image Differencing tool and Zonal statistics and interpreted as follows: negative change (subject to recurring moderate drought, moderate vegetation, precipitation, and temperature anomalies, urban expansion with a vegetation decrease), no change (areas with little or no change in vegetation values), positive change (containing positive precipitation and vegetation changes, and agricultural areas near flooded zones with a vegetation increase).

The flowchart below (Fig. 2) outlines the methodology followed to determine the LULCC dynamics and their impact on land degradation.

## Results

### LULC classification accuracy

Results show that LULC maps had an overall classification accuracy greater than 85% and a Kappa coefficient equal or greater than 0.82 (Table 3), except for the 1995 dry season and the 1999 wet season, which had overall accuracies of 84.07% and 84.81%, respectively. These were reasonably good overall accuracies as per the Manandhar et al. (2009) recommendation of at least 85%.

**Table 3:** Summary of wet and dry season LULC accuracy assessments

Year	Classified image	Kappa coefficient	Overall accuracy (%)
1990	Wet	0.85	87.04
	Dry	0.87	88.15
1995	Wet	0.85	87.41
	Dry	0.82	84.07
1999	Wet	0.82	84.81
	Dry	0.85	87.41
2005	Wet	0.83	85.19
	Dry	0.82	84.38
2010	Wet	0.85	87.04
	Dry	0.85	86.30
2015	Wet	0.85	87.04
	Dry	0.86	87.41
2019	Wet	0.86	87.41
	Dry	0.85	86.67

### LULC maps for wet and dry season

**Fig. 3:**
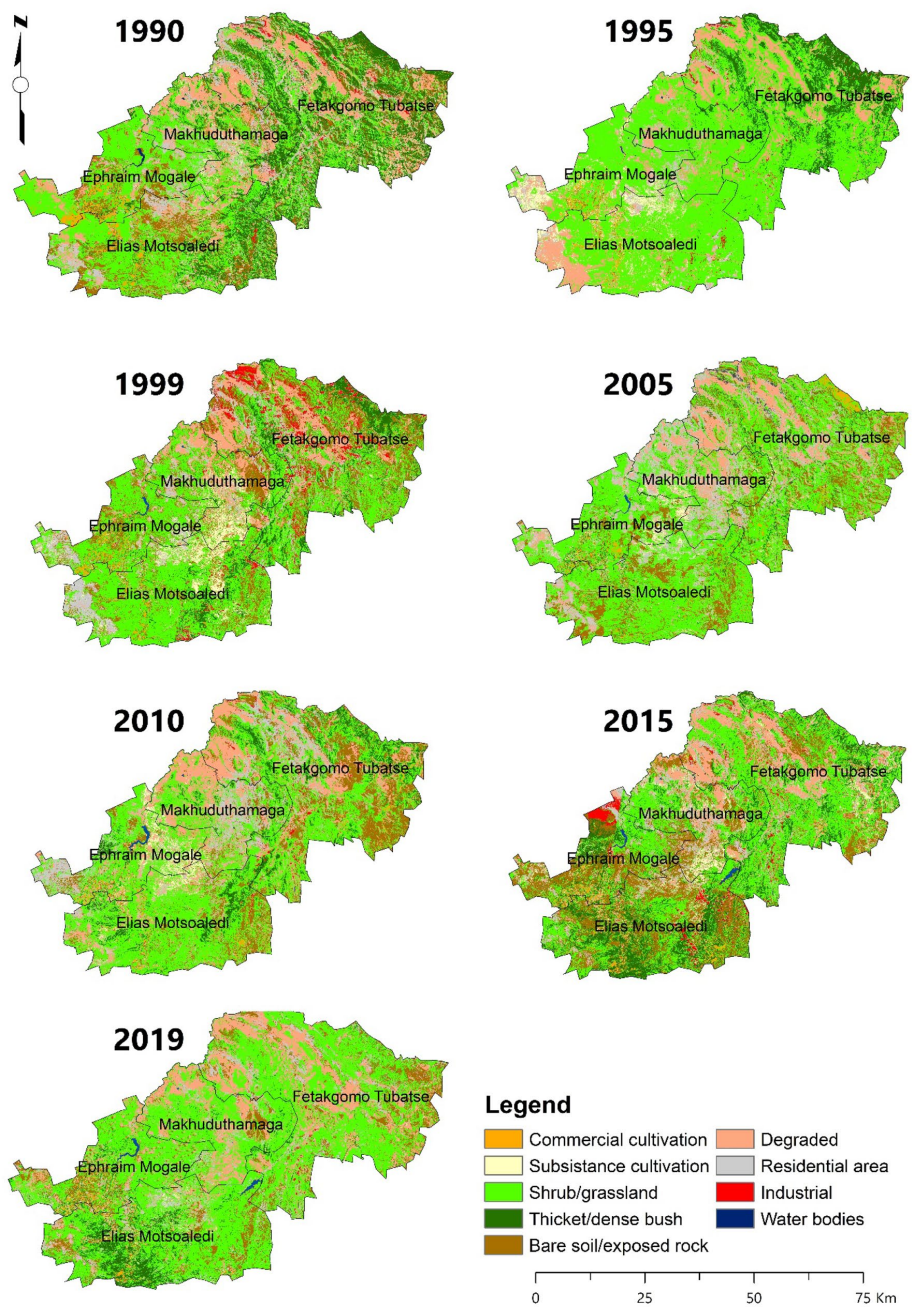
Five-year interval wet season LULC of the Greater Sekhukhune District Municipality from 1990 to 2019, mapped from relevant Landsat scenes

**Fig. 4:**
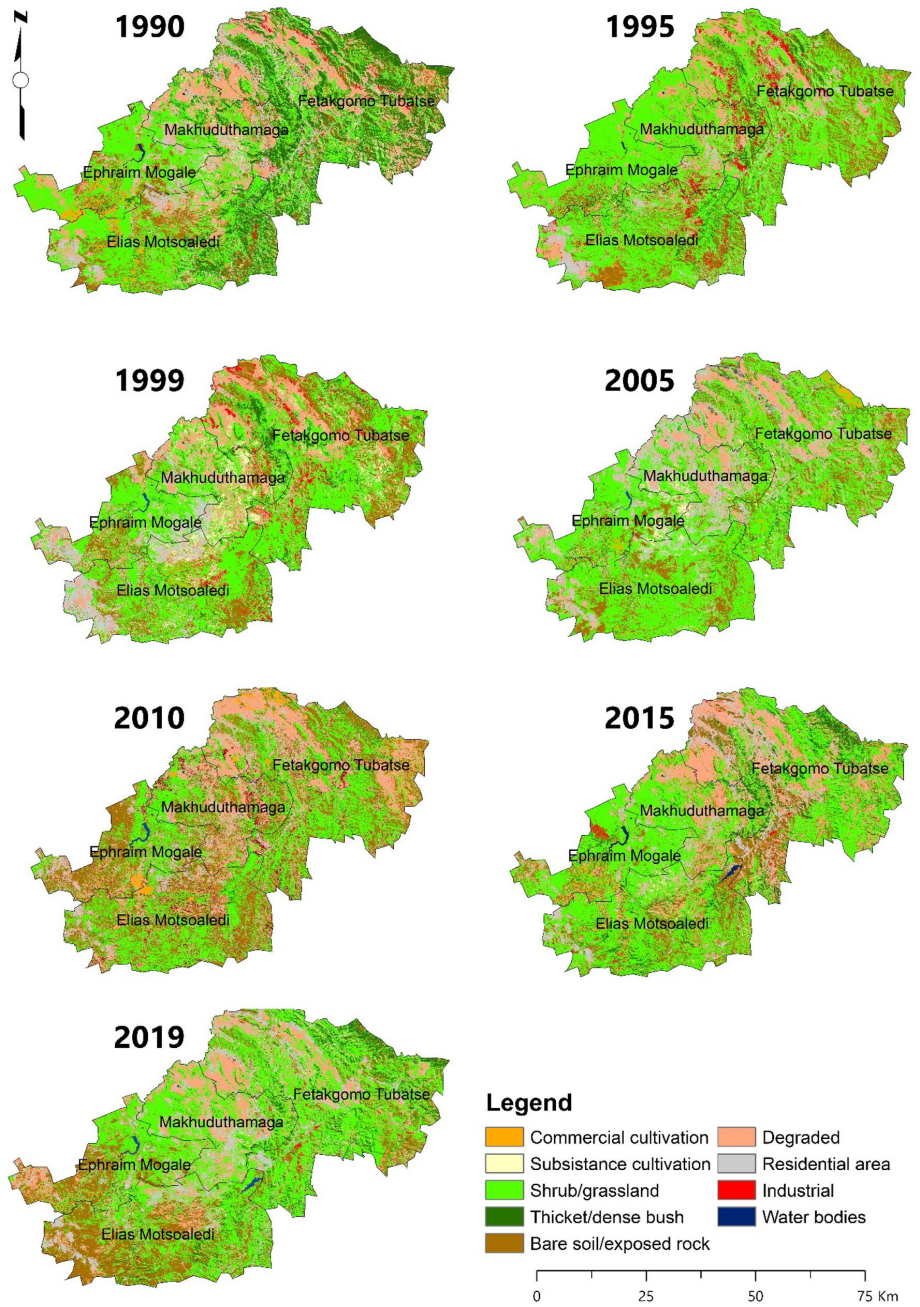
Five-year interval dry season LULC of the Greater Sekhukhune District Municipality from 1990 to 2019, mapped from relevant Landsat scenes

LULC classes were mapped for both the dry and the wet seasons at 5-year intervals (Fig. 3 and Fig. 4). Identified classes included commercial cultivation, subsistence cultivation, shrub/grassland, thicket/dense bush, bare/exposed soil, eroded land, residential, mines and quarries, and water bodies.

The LULC classification results indicate that shrub/grassland remains the dominant land cover and is spread throughout the district, while commercial cultivation is the main land use in the south to southeast sides of the district. The second dominant land cover is bare/exposed rock prevalent in the southern part of the district. LULCC dynamics show that in the wet season of 2015, an additional 21.06% (284,887.37 Ha) of land was bare and exposed, increasing the susceptibility of land to erosion. The third most dominant land cover in wet seasons is eroded land, which is prevalent in the central to northern parts of the district (in the Fetakgomo Tubatseand Makhuduthamaga local municipalities). These local municipalities were characterized by low-lying areas and plains (i.e. level plains with some relief and plains with open high hills or ridges), hence the presence of donga and gully features associated with significant water erosion, typically stream and flow line activities.

More land was eroded in 2015, reaching 20% (270,512.17 Ha) of the total area. Another indicator of a degrading land is that after 1995, thicket/dense bush in the district decreased significantly in dry season and was progressively converted to shrub/grassland.

### Evaluation of LULCC influence on LD: land degradation indicators

#### LULC changes

Area change, rate of change, and land conversions were used to identify change and determine LD. Over the past 30 years, the following increases were observed in the district during the wet seasons: a 98% increase in water bodies, a 76% increase in settlements (residential areas), and a 53% increase in shrub/grassland. The increase in water bodies is mainly attributed to the construction of De Hoop dam completed in 2014 (13^th^ largest in South Africa) on the Steelpoort River located in Fetakgomo Tubatse local municipality and covering 1690 Ha (Profection Design, 2016; GSDM IDP, 2018). However, field work observations indicated that the area of naturally occurring water bodies such as rivers and wetlands as well as vegetation has declined. The LULCs with the most significant decline are mine/quarries (81%), subsistence cultivation (80%), and thicket/dense bush (69%) in wet seasons. The decline in mine/quarries land use is attributed to the decline in operational mines where 18 out of 27 mines are non-operational.

**Fig. 5:**
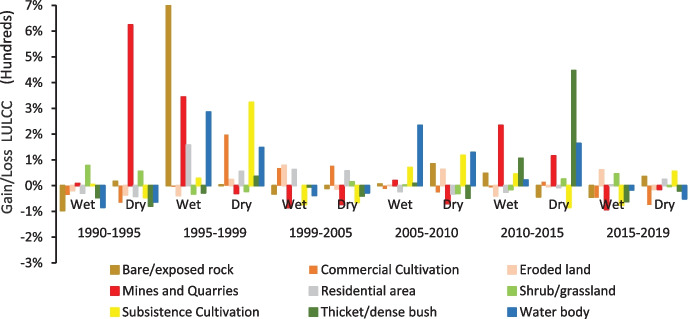
LULC percentage change-gain and loss during the study period

The changes in the area from 1990 to 2019 are illustrated in Fig. 5, with the most significant changes occurring from 1990 to 1995, and 2010 to 2015. Shrub/grassland changed to bare/exposed rock areas, an increase of 33% from the 1995 to 1999 in wet seasons. Eroded land increased in area by 74% from 1999 to 2005 in wet seasons and 63% in dry seasons between 2005 and 2010. This was a conversion of 79,494.00 Ha from shrub/grassland to eroded land.

**Fig. 6:**
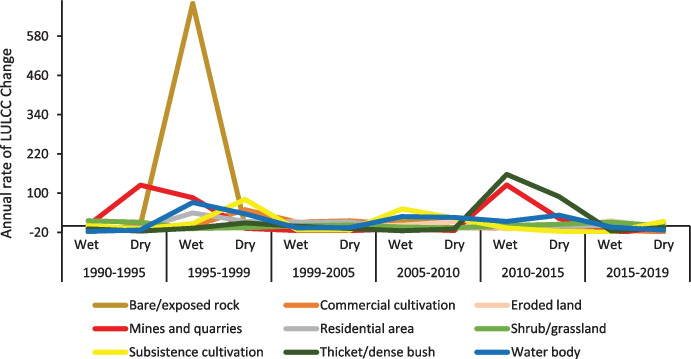
Annual rate of LULC change in LULC classes for wet and dry season

The rate of change of LULC is represented by the change of hectare/year for each class (Fig. 6). The highest declining rate of change per year was mines and quarries (16.12%), subsistence cultivation (15.50%), thicket/dense bush (13.30%), and commercial cultivation (11.13%) in the wet seasons between 1990 and 2019. Water bodies, residential areas, and shrub/grassland increased by 19.68%, 15.2%, and 10.66% annually, respectively. Bare/exposed rock increased by 10.49% per year. The spike increases in percentage gain and rate of bare/exposed rock from 1995 to 1999 is attributed to recurrent drought conditions recorded in the district in the 1990s as reported by Mpandeli et al. (2015).

The spatio-temporal changes over the 5-year intervals indicate a 13.71% annual decrease in commercial cultivation, mostly between 1990 and 1995 and subsequent annual decreases of 15.04% between 2015 and 2019. The area under subsistence cultivation decreased from 1999 at an annual rate of 14.87% from 1999 to 2005 and a subsequent annual decrease of 16.73% from 2010 to 2015. The area experienced a 31.62% annual increase in residential area from 1995 to 2005 and again by 6.86% between 2015 and 2019. Thicket/dense bush declined between 1990 and 1995 by an annual rate of change of 15.81% in the dry seasons and 2005 to 2010 by 14.95% in the wet seasons.

The land use land cover conversions also reveal that land productivity is declining (Table 4). LULC conversion was conducted using the transition matrix and the 14 most common conversions are shown in Table 4 for their respective seasons. The highest conversion was from shrub/grassland to bare/exposed rock by 129,255.85 Ha in the 2015 to 2019 dry season followed by thicket/dense bush to shrub/grassland by 110,625.63 Ha in 2010 to 2015 for the wet season and shrub/grassland to bare/exposed rock by 109,736.63Ha between 2010 and 2015 in the wet seasons. These highest conversions, and other conversions shown in Table 4, reveal that the productivity of the ecosystem in the district is decreasing as forested land is declining and being replaced mainly by shrub/grassland and subsequently bare/exposed rock and residential areas.

**Table 4:** Fourteen most common LULC conversion, period, and season

Rank	From class name	To class name	Period	Season	Area (Ha)
1	Shrub/grassland	Bare soil/exposed rock	2015–2019	Dry	129,255.85
2	Thicket/dense bush	Shrub/grassland	2010–2015	Wet	110,625.63
3	Shrub/grassland	Bare soil/exposed rock	2010–2015	Wet	109,736.63
4	Shrub/grassland	Bare soil/exposed rock	1995–1999	Dry	92,186.56
5	Shrub/grassland	Eroded Land	2005–2010	Dry	79,494.00
6	Thicket/dense bush	Bare soil/exposed rock	1990–1995	Dry	76,749.93
7	Eroded Land	Shrub/grassland	2015–2019	Dry	74,632.24
8	Residential	Shrub/grassland	2005–2010	Wet	73,953.02
9	Bare soil/exposed rock	Shrub/grassland	2015–2019	Dry	71,890.83
10	Bare soil/exposed rock	Shrub/grassland	2010–2015	Wet	70,188.57
11	Bare soil/exposed rock	Shrub/grassland	2005–2010	Wet	69,619.38
12	Bare soil/exposed rock	Shrub/grassland	1990–1995	Dry	69,079.58
13	Shrub/grassland	Bare soil/exposed rock	2005–2010	Wet	65,193.11
14	Shrub/grassland	Residential	1995–1999	Dry	64,465.42

#### NDVI change

**Fig. 7:**
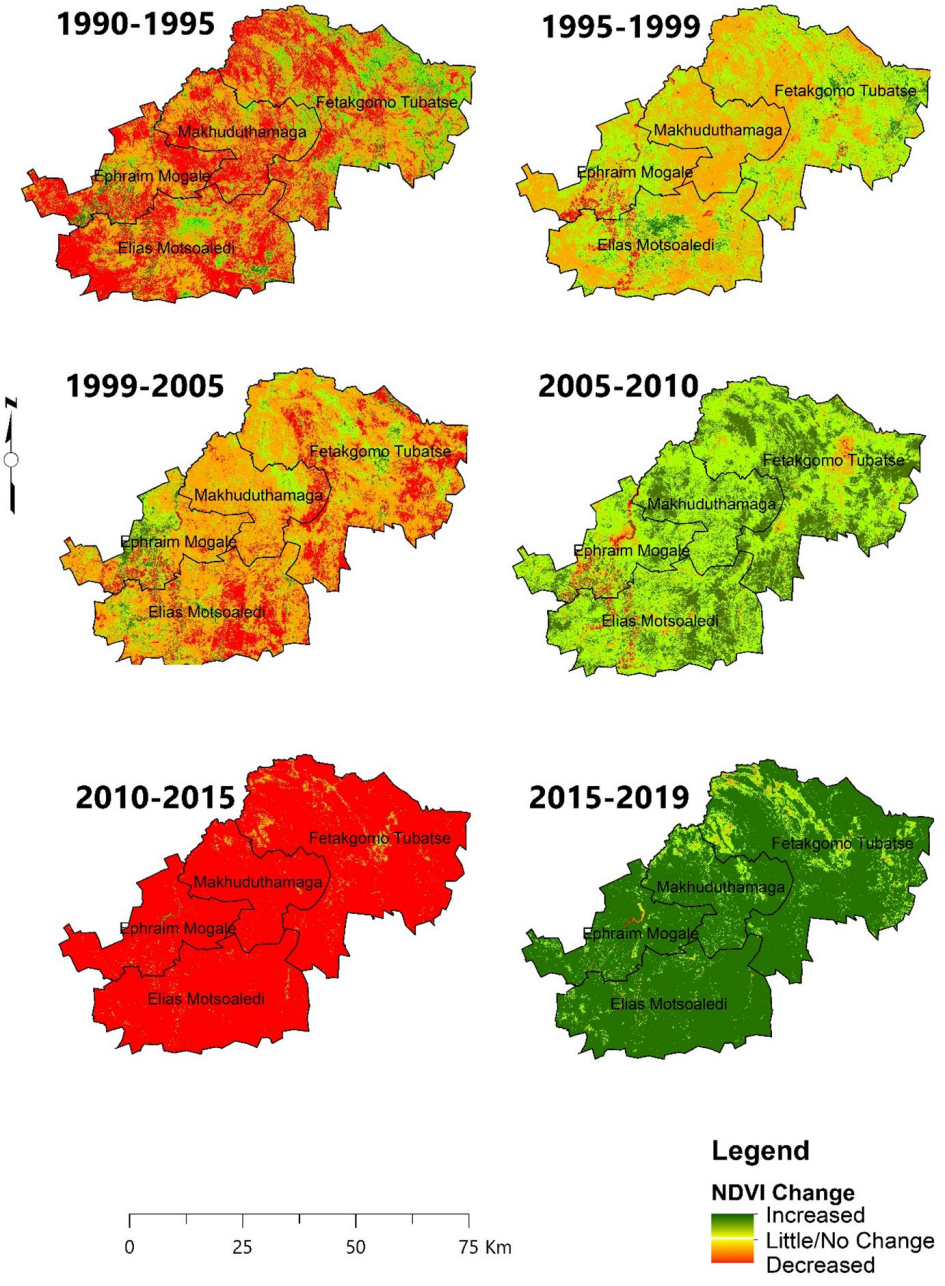
Wet season NDVI changes using image differencing using NDVI from 1990 to 2010

**Fig. 8:**
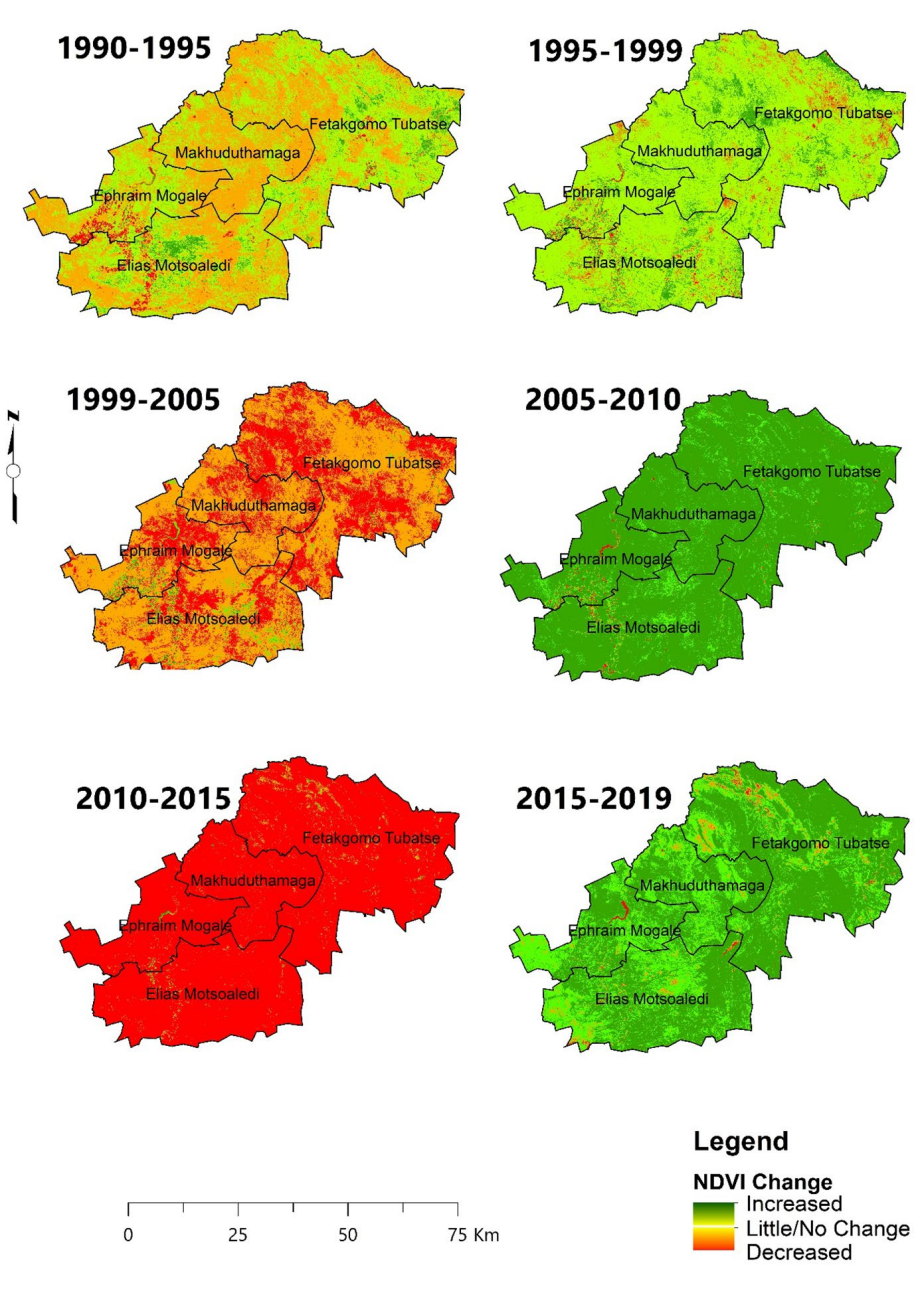
Dry season NDVI changes using image differencing

**Fig. 9:**
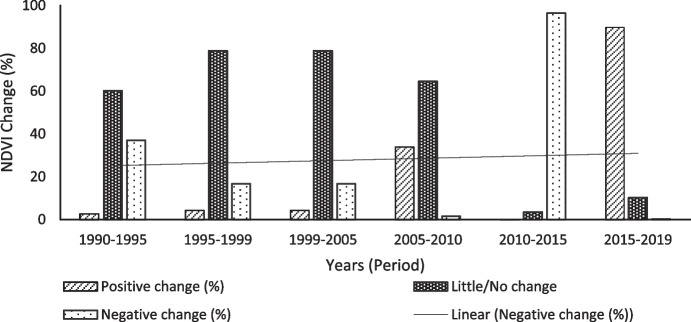
Wet season NDVI trends from 1990 to 2019

**Fig. 10:**
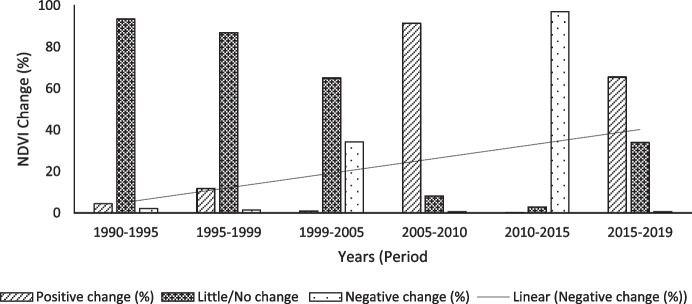
Dry season NDVI trends from 1990 to 2019

The second indicator of LD is land productivity, i.e. NDVI, shown in Figs. 7 and 8 and trends in Figs. 9 and 10, for the period between 1990 and 2019 at 5-year intervals. There was an increasing negative NDVI change in both seasons, with a steeper trend in the dry season. The wet and dry seasons show similar trends in productivity and indicate that the productivity of the area declined from 1990 to 2005 and started to increase between 2005 to 2010 and 2015 to 2019. Wet seasons recorded higher negative changes in 1990 compared to the dry seasons, while the dry seasons recorded higher negative changes between 1999 and 2005.

The district faced an extreme decline in productivity between 2010 and 2015, for both wet and dry seasons with the highest NDVI negative changes of 96.39% and 97.05%, respectively. The LULC conversion shows that the second most common conversion was recorded between 2010 and 2015, when thicket/dense bush was converted to shrub/grassland, then subsequently shrub/grassland replaced with bare/exposed rock. In 2019, for both wet and dry seasons, there was an increase in NDVI.

### Potential driving factors of LULCC and LD in the district: linking RS results (LULCC and NDVI) and key-informant interviews and tribal council workshop results

As mentioned above, a semi-structured questionnaire was used to interview key informants, i.e. natural resource managers, crop production, animal production, and extension services officials per local municipality from the Limpopo Department of Agriculture and Rural Development (LDARD). The interviews revealed that the main drivers of LULC changes contributing to LD were soil erosion, an increase in bare soil due to overgrazing and lack of grazing management, cropland abandonment, settlement encroachment into productive cropping land, policy and institutional changes, wood harvesting, and the land tenure system.

#### Soil erosion and increase in bare soil cover

**Fig. 11:**
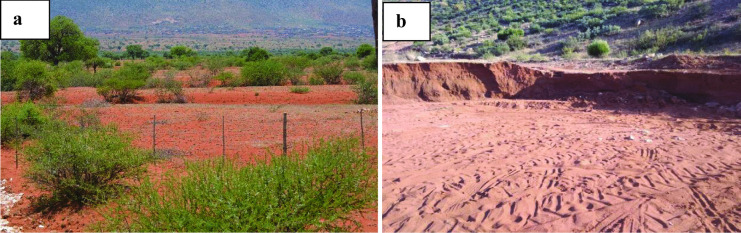
**a** Overgrazing and bush encroachment into abandoned cropping fields; (**b**) illegal sand mining in a gully in Mphanama village, Fetakgomo Tubatse municipality

The interviews highlighted that soil erosion in the area is mainly due to human-induced activities exacerbated by flash floods. Overgrazing was noted as the main contributor of increased eroded land and bare soil because of uncontrolled/lack of rotational grazing (Fig. 11a). All key informants noted that grazing capacity has also been reduced due to inappropriate and/or lack of grazing management such as rotational grazing. Overstocking and vandalized fences have contributed to land degradation. Also, illegal sand mining was noted as one of the contributors to soil erosion as natural vegetation is removed and existing gullies are extended due to the activity (Fig. 11b).

#### Communal rangeland land use changes: settlement encroachment into cropping land, cropland abandonment, and bush encroachment

The key informants noted several land use changes in the rangeland that affected the ecosystem and land productivity. An increasing population has led to an increase in built-up residential areas which encroach into cropping lands (encroaching into communal rangeland). This has resulted in a decline in croplands and crop output. Abandonment of croplands was reported by all informants to be widespread and was mainly attributed to unpredictable rainfall, lack of interest in agriculture as a livelihood, a growing crisis of an ageing farmer population without replacement, migration, social grants as a major source of income, and improper cropping methods. The key informants also noted that there is prevalent bush encroachment which reduces vegetation cover and livestock carrying capacity.

## Discussion

This study explored the impacts of LULC change on land degradation in the pre-dominantly rural Greater Sekhukhune District Municipality under a dual land use administration system. The study also analyzed the spatio-temporal changes over 5-year interval during the wet and dry seasons to determine periods of land use changes, understand the main driving mechanisms, and assess ecosystem recovery. Overall, the district experienced a significant reduction of thicket/dense bush to shrub/grassland, which consequently changed to bare and eroded lands. These changes were mainly attributed to injudicious rangeland management, land use administration challenges, and rainfall variability.

Results showed that significant amounts of thicket/dense bush cover were reduced to shrub/grassland after 1995, attributed to the deterioration and erosion of topsoil. Since the area is within the savanna biome, it has a high tendency of crusting that facilitates high runoff and erosion that depletes topsoil organic matter (Mills & Fey, 2004). The poor soils induce the generation of hardy shrubs that can survive on lower quality soils (Jacobs, 2000). Another reason for the significant decline of thicket/dense bush and other classes such as subsistence and commercial cultivation is that the vegetation and soil productivity is often unable to recover after dry seasons due to prolonged drought and/or anthropogenic activities. Murray-Tortarolo et al. (2016) report that changes in water availability over the dry season affect vegetation productivity throughout the year, driving changes in regional NPP even in the wet season. Between 2010 and 2015, there was a decline in forest, shrubland, and cropping land. This decline is attributed to prolonged droughts that occurred from 2010 to 2015 as reported by Mpandeli et al. (2015) and Meza et al. (2021). Other reasons for a reduction of subsistence cultivation are the abandonment of cropland due to declining land productivity and increased reliance on government grants as noted by Sinyolo et al. (2017).

As shown by the LULC changes of the study area, frequent prolonged drought events, particularly in rangelands of semi-arid regions, have detrimental effects on the natural and socio-economic productivity of the landscape. The frequent prolonged droughts reduce vegetation cover, increase bare soil, and expose the soil to erosion. Moreover, fire occurrence due to frequent and prolonged drought (Vetter, 2009) further reduces vegetation cover and the physical grazing carrying capacity. In agreement with Mpandeli et al. (2015), our results show a synergistic effect of prolonged drought and intensive rainfall events that lead to LULC changes and land degradation in the district. As noted by Mohamadi and Kavian (2015), alternating prolonged drought events and intensive rainfall in the district further result in landscape degradation.

Bush encroachment was observed across the rangeland of the district and could be an indicator of prevalent land degradation. Bush encroachment reduces grass cover and livestock grazing capacity as vegetation suppresses palatable plant species and grasslands (Graw et al., 2017). Overstocking and reduced grazing land have further led to LD. Also, as noted by Bond et al. (2003), in semi-arid rangelands, elevated CO_2_ attributed to the changing climate may favour the growth of trees and shrubs in comparison to grasses. In areas that are heavily reliant on grazing as a major socio-economic activity, this may facilitate further degradation.

The increasing negative NDVI change in the wet and dry seasons in the study area is consistent with Kumar et al. (2015) and Murray-Tortarolo et al. (2016) who noted that the dry season ecosystem plays a critical role in landscape productivity. According to Murray-Tortarolo et al. (2016), areas subjected to recurrent moderate drought, precipitation, and temperature anomalies are more vulnerable to LD. Anthropogenic LD drivers such as overgrazing and unsustainable land use practices, e.g. unsustainable wood harvesting, are known to be the major causes of LD in communal rangelands (Hoffman & Ashwell, 2001); hence, it is important to explore and document these factors as a first step to achieving Land Degradation Neutrality (UNCCD, 1994). Furthermore, studies have shown that cropland abandonment and overgrazing promote bush encroachment, which in turn reduces grazing land, hence further feeding into a cyclic loop of LD (Buitenwerf et al., 2012; Graw et al., 2016; Stephens et al., 2016; Stevens et al., 2017).

The key-informant interviews and workshop with the tribal authority revealed that policy changes, land tenure conflicts, and uncoordinated land use plans are contributing to land use changes that degrade the land. This mostly took place after 1998 when most policies and institutional changes were implemented following their introduction post-1994. The key informants emphasized that the phasing out of the rangers who used to enforce local grazing management decisions, unsustainable wood harvesting and overall injudicious rangeland, and lack of accountability and coordinated communal land management have all led to the deteriorating landscape. Generally, communal land has been perceived as vulnerable due to the presumed inability of land users to make collective decisions for the sustainable management of common resources. Key informants highlighted that individual users act independently for self-interest and contrary to the common good, hence causing depletion of resources. This is a reflection of the concept of the “tragedy of commons” paradigm (Hardin, 1968). The absence of rangeland management institutions in the district has also resulted in the vandalism of erosion control structures and theft of fences that control animal movement as a form of rangeland management in communal lands (Herd-Hoare, 2020).

Abandonment of cropland has increased significantly in the district, partly due to free roaming animals in various villages that has discouraged subsistence farmers to continue crop cultivation. Land tenure, particularly in Ephraim Mogale local municipality, is the main cause of cropland abandonment and degradation. Key informants highlighted that land conflicts have led to more land lying fallow due to land claims and a lack of capital after the land redistribution that occurred after 1998. Forested land has declined due to unsustainable wood harvesting throughout the years and has been converted to shrub/grassland cover. However, despite these communal rangeland management setbacks, a legal system through traditional councils has been enabled to play an increasingly important role in the local administration of communal areas since the introduction of the Communal Land Act in 2004 (Ntsebeza 2005; Republic of South Africa, 2004).

## Conclusion

This study sought to assess the LULCC in GSDM and its impacts on land degradation. Specifically, the study sought to determine drivers of land degradation in a traditional rural district with dual land use systems within a semi-arid environment. The findings of the study reveal that the district is slowly changing from a savannah biome to a grassland, and this has severe impacts on local livelihoods, particularly pastoralists and rain-fed farmers. Furthermore, bush encroachment and plant species invasion are major concerns in the district’s communal rangeland as they reduce existing grazing land. Findings typify the situation in most of the former “homelands” in South Africa. Hence, this study serves as a basis to support multi-stakeholder cooperation in efforts to mitigate LD. Although efforts have been made to control grazing, animal movement and erosion control structures, vandalism, and a lack of accountability from the communities and their leadership remain serious challenges. Key informants emphasized the need for transparency in land use management to mitigate land degradation. Hence, the study highlights the need for cooperation between government, tribal authorities, and land users in protecting natural resources and mitigating land degradation. Accordingly, land management policies need to focus on an integrated and coordinated approach and awareness to focus on land use and land care.

## Supplementary Information

Below is the link to the electronic supplementary material.

Supplementary file1 (DOCX 19 KB)

## Data Availability

The data that support the findings of this study are available on request from the corresponding author. The data are not publicly available due to privacy or ethical restrictions.
