# Supplementary material for: Impact of land use and land cover change on land degradation in rural semi-arid South Africa: case of the Greater Sekhukhune District Municipality
Source: Environ Monit Assess. 2023 May 23;195(6):710. doi: 10.1007/s10661-023-11104-0 (PMC10205882; doi:10.1007/s10661-023-11104-0)
Supplement: Supplementary file 1 — Supplementary file1 (DOCX 19 KB) [file 10661_2023_11104_MOESM1_ESM.docx]

# Appendix

Key informant Semi-structured interview Questionnaire

*Evaluation of Land Use Land Cover Change influence on LD*

| **Local municipality:** | Greater Fetakgomo/ Tubatse | Elias Motswaledi | | Ephraim Mogale | Makhudumathamaga | |
| --- | --- | --- | --- | --- | --- | --- |
| **Key Informant:** | Natural resource manager | | Extension officer | | | Crop production |

| **How long have you been working in the area? In the years worked, what are the most significant changes experienced or seen in the**   1. **(e.g. lower yields, increase in gullies, change in grazing species composition, invasive species, less palatable species, lower groundwater table etc.)?** | | | | |
| --- | --- | --- | --- | --- |
|  | | | | |
| 1. **Where did these changes occur and why in those particular locations?** | | | | |
|  | | | | |
| 1. **When did the changes occur and why then? what triggered those changes** | | | | |
|  | | | | |
| 1. **What do you think are the (i) direct causes and (ii) indirect causes are the main reasons for changes in LULCC?** | | | | |
| 1. **Direct causes** 2. **Indirect causes** | | | | |
| 1. **Impact of changes in LULCC on land degradation? Indicate where applies the causes of land degradation and specify** | | | | |
| **Direct causes** |  | | **Specify** | |
| (i) Inappropriate soil management |  | | | |
| (ii) Inappropriate crop and rangeland management (annual, perennial, shrub and tree crops) |  | | | |
| (iii) Deforestation and removal of natural vegetation due to: |  | | | |
| (iv) Disturbance of the water cycle leading to accelerated changes in the water level of groundwater aquifers, lakes and rivers (improper recharge of surface and groundwater) due to: |  | | | |
| (v) Natural causes: i.e. heavy or extreme rainfall, change of seasonal rainfall (perception of land users) |  | | | |
| (vi) Others |  | | | |
| **Indirect causes** | **Specify** | | | |
| (i) Population pressure |  | | | |
| (ii) Land Tenure: Poorly defined tenure security |  | | | |
| (iii) Poverty: limits land-user investment and choice. Poor people often have no alternative but to use marginal land that may be particularly prone to land degradation (e.g. steeply sloping areas |  | | | |
| (v) Labour Availability: Shortage of rural labour (e.g. through migration, the prevalence of diseases, out-migration, aging) can lead to the abandonment of traditional resource conservation practices such as terrace maintenance |  | | | |
| (vi) Inputs and infrastructure: (roads, markets, Co-operatives, fencing to manage animal movement etc.): |  | | | |
| (vii) Informal institutions (gi): local rules and regulations, social and cultural arrangements & obligations affecting access to resources. |  | | | |
| (viii)Others |  | | | |
| 1. **What are the potential economic, social and environmental impacts of LULCC changes and land degradation?** | | | | |
|  | | | | |
| 1. **What is done to address these changes? What are the methods used to improve soil fertility, reduce erosion, and manage water resources? Has there been the adoption of new practices and/or changed your management patterns?** | | | | |
| **If adoption of SLM practices** | | **If no adoption of SLM practices** | | |
| 1. Is the measure used to prevent, reduce degradation or rehabilitate degraded lands? | | 1. What are the constraints for adoption e.g. insecurity of tenure, seasonal migration, land shortage, lack of capital, labour unavailability)? | | |
| 1. Are the new practices effective? | |  |  |  |
| 1. What is the percentage of farmers using these practices? | |  |  |  |
| 1. Other | |  | | |
| 1. **Are there protected areas and why are they protected?**   **Areas once heavily utilized may have become protected, preventing the harvesting of forest products, and use for grazing. What impact has this had on the land and users’ livelihoods?** | | | | |
|  | | | | |
| 1. **What mechanisms are used to control the use of land such as grazing periods?** | | | | |
| **Formal regulations** | | | | **Informal (customary) regulations** |
|  | | | |  |
| 1. **Are there any land-use conflicts between the two systems? If so what are the conflicts and how can they be harmonised? i.e. access, use, and right to land** | | | | |
|  | | | | |
